# Current Understanding of the Correlation of Lignin Structure with Biomass Recalcitrance

**DOI:** 10.3389/fchem.2016.00045

**Published:** 2016-11-18

**Authors:** Mi Li, Yunqiao Pu, Arthur J. Ragauskas

**Affiliations:** ^1^BioEnergy Science Center, Biosciences Division, Joint Institute of Biological Science, Oak Ridge National LaboratoryOak Ridge, TN, USA; ^2^Department of Chemical and Bimolecular Engineering, University of Tennessee KnoxvilleKnoxville, TN, USA; ^3^Department of Forestry, Wildlife, and Fisheries, Center for Renewable Carbon, University Tennessee Institute of AgricultureKnoxville, TN, USA

**Keywords:** biomass recalcitrance, lignin structure, cell wall, pretreatment, enzymatic hydrolysis

## Abstract

Lignin, a complex aromatic polymer in terrestrial plants, contributes significantly to biomass recalcitrance to microbial and/or enzymatic deconstruction. To reduce biomass recalcitrance, substantial endeavors have been exerted on pretreatment and lignin engineering in the past few decades. Lignin removal and/or alteration of lignin structure have been shown to result in reduced biomass recalcitrance with improved cell wall digestibility. While high lignin content is usually a barrier to a cost-efficient application of bioresources to biofuels, the direct correlation of lignin structure and its concomitant properties with biomass remains unclear due to the complexity of cell wall and lignin structure. Advancement in application of biorefinery to production of biofuels, chemicals, and bio-derived materials necessitates a fundamental understanding of the relationship of lignin structure and biomass recalcitrance. In this mini-review, we focus on recent investigations on the influence of lignin chemical properties on bioprocessability—pretreatment and enzymatic hydrolysis of biomass. Specifically, lignin-enzyme interactions and the effects of lignin compositional units, hydroxycinnamates, and lignin functional groups on biomass recalcitrance have been highlighted, which will be useful not only in addressing biomass recalcitrance but also in deploying renewable lignocelluloses efficiently.

## Introduction

**Figure 1:**
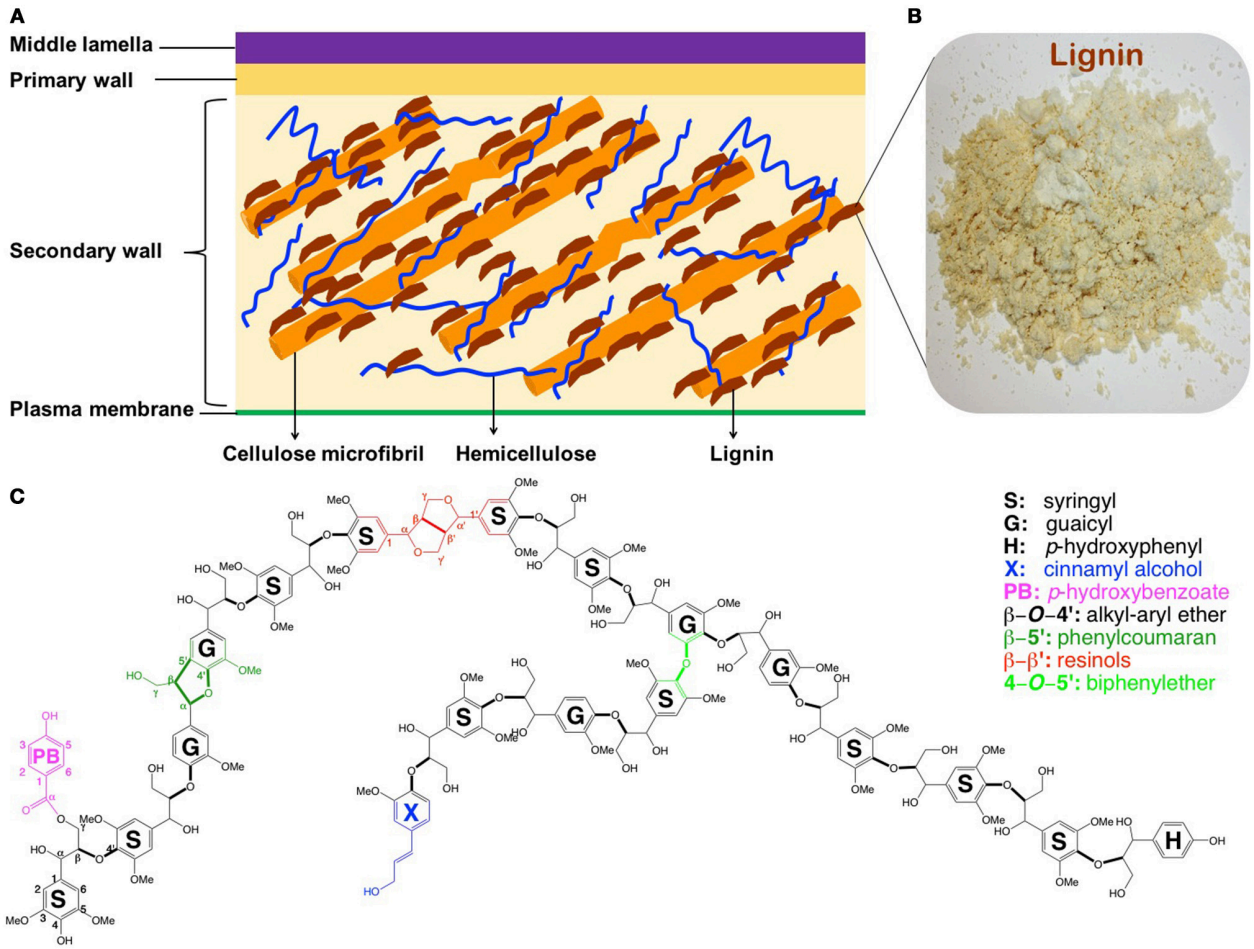
**Simplified structure of plant cell walls (A)** (Phitsuwan et al., 2013), lignin isolated from poplar **(B)**, and schematic structure of poplar lignin **(C)** (Vanholme et al., 2010).

The conversion of renewable lignocellulosic biomass to fuels and valuable co-products, usually referred as biorefining, has been advanced recently (Ragauskas et al., 2006). However, transition from fossil-based to biomass-based products using current feedstocks and technologies has been challenged by the inherent resistance of plant cell walls to microbial and enzymatic deconstruction, namely, recalcitrance (Himmel et al., 2007). While other factors could not be neglected, the presence of lignin (ca. 15–35% by weight) is one of the most significant recalcitrance contributors, which escalates the processing costs (i.e., necessitated pretreatment and enlarged enzyme amount; Mosier et al., 2005; Pu et al., 2011, 2013). Lignin is an amorphous and complex aromatic polymer providing terrestrial plants mechanical support, stress response, pathogen resistance, and water transport (Boerjan et al., 2003). By bonding or embedding with other biopolymers (cellulose and hemicellulose), lignin strengthens the integrity and rigidity of the plant cell wall yielding a complex macro-molecular assembly (Figure 1). This lignin-polysaccharides matrix renders cell walls recalcitrance for biorefining. Depending on biological species, lignin in plants derives primarily from three phenylpropanoid monolignols—*p*-coumaryl, coniferyl, and sinapyl alcohols (Li M. et al., 2016; Yoo et al., 2016b), which give rise to *p*-hydroxyphenyl (H), guaicyl (G), and syringyl (S) units, respectively (Figure 1). The inter-linkages between these subunits are β-*O*-4′, β-5′, α-*O*-4′, 4-*O*-5′, β-β′ in common, and β-1′, and 5-5′ in minor amount.

In biomass, lignin content, structure, subunits, and linkages with polysaccharides own their remarkable importance to cell wall recalcitrance, usually gauged by biomass enzymatic hydrolyzability (Zeng et al., 2014; McCann and Carpita, 2015). Lignin limits the enzymatic hydrolysis of biomass through two primary mechanisms: restricting polysaccharides accessibility (physical barrier and/or lignin-carbohydrate complexes) and non-productive binding with enzymes (inhibition).

Current strategies to reduce lignin involved biomass recalcitrance mainly include (i) pretreatment technologies (Mood et al., 2013; Pu et al., 2013), (ii) perturbing lignin biosynthesis (Bonawitz and Chapple, 2010; Simmons et al., 2010), and (iii) enzyme engineering and modification (Güven et al., 2010). Whichever strategy is used, fundamental understanding of the influence of lignin's physicochemical properties on biomass recalcitrance is crucial to advance the biologically-based biorefinery. This mini-review aims at recent findings on the relationship between lignin structure and biomass recalcitrance.

## Lignin during pretreatment

Lignin restricts enzymatic hydrolysis commonly through physical barrier and non-productive/non-specific binding to enzymes. While many pretreatments (e.g., alkaline/organosolv) substantially remove lignin yielding biomass with enhanced cellulose accessibility, some thermochemical pretreatments such as HWP, DAP, SEP, and AFEX, usually with limited delignification, lead to reduced biomass recalcitrance too (Mood et al., 2013; Pu et al., 2015). The cell wall matrix disruption could be facilitated by cleavage of aryl ether linkages in lignin and LCC linkages, which increases pore volumes and cellulose accessibility. For example, β-*O*-4′ linkages are significantly cleaved in HWP poplar (Samuel et al., 2013) and DAP switchgrass (Samuel et al., 2010). AFEX reduced lignin-hemicellulose ester linkages revealed by a substantial loss of ferulate groups in the residual lignin (Chundawat et al., 2011). In addition, the translocation and redistribution of lignin coalescing into lignin droplets of various morphologies have been found in corn stem and switchgrass during DAP and HWP, which related to increased cell wall accessibility (Donohoe et al., 2008; Pingali et al., 2010). It was found that the lignin-hemicellulose complex underwent phase separation during pretreatment which drove the lignin aggregation followed by collapse, leading to increasing cell wall porosity (Langan et al., 2014). Using mixed poplar and Avicel, the lignin droplets from poplar was redeposited on the Avicel during HWP, causing 30–40% decrease of initial hydrolysis compared with Avicel control (Li H. et al., 2014). Interestingly, lignin-like droplets, pseudo-lignin, which inhibit enzymatic hydrolysis, can form from carbohydrates during acidic pretreatments (Sannigrahi et al., 2011; Hu et al., 2012).

## Lignin-enzyme interactions

Lignin has remarkable influence on enzymatic performance owing to lignin-enzyme interactions. Stemming primarily from hydrophobic, ionic, and hydrogen bond interactions with protein, lignin-enzyme interactions are highly influenced by the physicochemical properties of lignin (Nakagame et al., 2011a). Lignin is generally found inhibitory to cellulose hydrolyzability due to non-productive/non-specific binding with enzymes. For example, when added into the enzymatic hydrolysis of Avicel or pretreated biomass, several commercial lignins and isolated lignins from variable biomass origins apparently decreased saccharification (Nakagame et al., 2010, 2011c; Kim, 2012; Ko et al., 2015). However, cellulolytic lignin residues from corn stover and wheat straw had minimal impact on the hydrolyzability of pretreated biomass (Barsberg et al., 2013). Surprisingly, recent studies reported certain technical lignins with promoting effects, e.g., lignosulfonate (Wang et al., 2013; Zhou et al., 2013), organosolv lignin (Lai et al., 2014), Kraft lignin (Wang et al., 2015), and alkali lignin (Li Y. et al., 2016). These authors attributed the positive effect of lignin to the alleviation of non-productive binding *via* lignosulfonate-enzyme complex or surfactant protection. Several studies also suggested that lignins isolated from herbaceous plants had relatively less inhibition than woody biomass, likely because (i) branched lignin (G-lignin) is more inhibitory than linear lignin (S-lignin) and (ii) the formation of metal ion (e.g., Ca^2+^)-lignin complex could reduce lignin-enzyme interactions (Liu et al., 2010; Barsberg et al., 2013). In addition, the inhibitory effects of lignin depend on pretreatment severity. Lignin derived from more severely pretreated biomass exhibited more pronounced inhibition to the hydrolysis of Avicel because increased pretreatment severity resulted in more condensed structure (Nakagame et al., 2011c; Ko et al., 2015). Lignin repolymerization with increased C-C condensed structure presumably *via* the formation of carbonium ions can occur during HWP, DAP, and SEP (Pu et al., 2015). Lignin condensation associated with hydrophobicity influences lignin-enzyme interactions significantly. A few studies have shown that lignin with increased condensation from pretreated biomass tended to adsorb more enzymes, resulting in more inhibitory to cellulose hydrolysis (Yu et al., 2014; Ko et al., 2015; Huang et al., 2016; Yang and Pan, 2016).

Isolated lignin seems be more inhibitory to the hydrolysis of pure cellulose than lignocellulosic materials. Isolated Douglas-fir lignin decreased the hydrolysis yields of Avicel and pretreated softwood by 46 and 9%, respectively (Kumar et al., 2012). Kraft lignin and lignosulfonate inhibited pure cellulose saccharification but enhanced the hydrolysis of pretreated biomass (Liu et al., 2010; Kim, 2012; Zhou et al., 2013; Wang et al., 2015). In comparison to pure cellulose, the complexity of lignocellulosic substrates probably plays a role in the lignin-enzyme interactions. As noted by Zhou et al., lignosulfonate interacts with both the bound and soluble lignin of the substrate (Zhou et al., 2013). Hydrolysis of the same biomass with different degree of sulfonation demonstrated different enhanced digestibility when sulfonated lignin was added (Wang et al., 2015). Another important finding was that non-productive/non-specific binding predominated for less accessible biomass; with increased cellulose accessibility of lignocellulose, the inhibitory effects of lignin dwarfed (Kumar et al., 2012). Therefore, the lignin-enzyme interactions, conventionally termed as “detrimental effect,” varies significantly on lignin chemistry, type of substrate as well as pretreatment techniques employed.

## Lignin monolignol compositional units

The monolignol compositional units (relative abundance of H, S, and G) of lignin have been documented to affect biomass digestibility. Without pretreatment, several studies found that S/G ratio was negatively related to the enzymatic hydrolysis of untreated biomass, e.g., engineered poplar, eucalyptus mutants, and maize cell wall (Zhang et al., 2011; Papa et al., 2012; Min et al., 2013). The authors deduced the negative effect of S/G likely to a more efficient coverage of S-lignin (extended shape) than G-lignin (branching) on cellulose fibrils according to a proposed molecular model (Besombes and Mazeau, 2005a,b). However, a few other studies reported that the hydrolyzability of untreated biomass was not affected by S/G ratio, such as *Populus* natural variants with different S/G ratio (1.0–3.0) (Studer et al., 2011), high G (95%) and high S (91%) contained *Arabidopsis* (Li et al., 2010), and transgenic poplar lines with 87 and 93% S (Mansfield et al., 2012) showing basically similar hydrolysis efficiency vs. their corresponding controls. It seems that the small hydrolysis improvement in lower S/G plants without pretreatment might be influenced by factors arising from other cell wall components, variated lignin content, or type of enzyme.

By contrast, S/G ratio in lignin has shown remarkable influence in pretreatment and the hydrolyzability of pretreated biomass. Enhanced saccharification upon S/G ratio has been reported on *Populus* (Studer et al., 2011) and *Arabidopsis* (Li et al., 2010) by HWP, and poplar by SEP (Mansfield et al., 2012). S-unit was preferentially removed relative to G-unit in poplar during flowthrough pretreatment (Trajano et al., 2013). In addition, S/G ratio is positively related with delignification of hardwood by green liquor and Kraft pulping (Santos et al., 2011; Min et al., 2013). According to these authors, high S/G ratio benefits pretreatment mainly due to (i) lignin depolymerization: S-lignin features higher level of labile β-*O*-4′ linkages which are readily cleavable during pretreatment; and (ii) delignification: S-lignin with relatively higher occurrence of β-β′ bonds leads to lower molecular weight which could facilitate lignin migration and removal. However, inconsistent results have also been reported on the correlation of S/G ratio with saccharification efficiency, e.g., high S/G ratio was negatively associated with the saccharification of both NaOH and H_2_SO_4_ pretreated Miscanthus (Xu et al., 2012; Li M. et al., 2014) and green liquor pretreated wheat straw (Xu et al., 2012; Li M. et al., 2014; Jiang et al., 2016). An unclear correlation has also been reported, such as green liquor and Kraft pretreated Eucalyptus (Papa et al., 2012; Santos et al., 2012), NaOH and H_2_SO_4_ pretreated wheat and rice samples (Wu et al., 2013), and mild acid treated maize cell wall (Zhang et al., 2011). When the S/G ratio of the remaining lignin in pretreated biomass is studied, contradictory results also exist: a lesser proportion of non-condensed lignin in the pretreated biomass was reported to be beneficial to biomass hydrolysis as non-condensed lignin (high β-*O*-4′) tended to be linear shape, likely a higher coverage over cellulose fibers (Zhang et al., 2011; Yeh et al., 2014); by contrast, others found high S/G ratio of lignin in the pretreated biomass was positively correlated with enzymatic digestibility as branched G-lignin gave rise to more physical barrier (Santos et al., 2012; Yu et al., 2014). These inconsistent results suggest that S/G ratio contributes only partially to biomass recalcitrance, and the complexities of biomass species and pretreatment with concomitant other cell wall structure changes likely shelter the effects arising from lignin composition.

Due to the low occurrence of H-lignin reported in natural plants, the influence of H-lignin on biomass recalcitrance is relatively less investigated. Interestingly, a few recent studies revealed that high H-lignin contained plants exhibited reduced biomass recalcitrance. For instance, transgenic Alfalfa (50–76% H) had increased alkaline extractability of lignin-like content vs. control (5% H) (Ziebell et al., 2010); the H/G ratio measured in the KOH-extractable lignin of wheat and rice had a strong positive correlation with glucose yield of pretreated biomass (Wu et al., 2013); H-rich (89%) Arabidopsis mutant exhibited significantly higher sugar yield than G-rich (96%) and S-rich (92%) mutants without pretreatment (Shi et al., 2016); hexose yield increased upon H level in alkali pretreated Miscanthus (Li M. et al., 2014). The speculated reasons for the positive effect of H-lignin are (i) reduced lignin molecular weight, (ii) reduced cellulose crystallinity via H unit-glucan bonding; and (iii) higher linkage activities between H monomer than G and S.

## Hydroxycinnamates in lignin

Biomass recalcitrance is also related to the presence of hydroxycinnamates in lignin, mainly ferulates (FA) and *p*-coumarates (*p*CA) which are two important hydroxycinnamates in grass (Buranov and Mazza, 2008; Ralph, 2010). FA is reported to meditate the cross-linking of polysaccharides-polysaccharides and polysaccharides-lignin in cell wall (de O Buanafina, 2009; Azarpira et al., 2011). The presence of this cross-linking draws lignin to polysaccharides proximately resulting in an increased recalcitrance. Reduced FA-mediated cross-linking of lignin-polysaccharides in maize (Jung and Phillips, 2010) and silage (Jung et al., 2011) had improved digestibility. Due to the strong negative correlation of etherified phenols, FA content in forage crop silages was good predictor for *in vivo* cell wall digestibility (Taboada et al., 2010). To enhance biomass digestibility, one of the effects of pretreatment is to cleave FA cross-linkages and promote lignin degradation and coalescence (Li et al., 2012; Qin et al., 2015; Martínez et al., 2016; Yoo et al., 2016a). Rather than cross-linking inter-components in cell wall, *p*CA are usually esters pendantly linked on lignin (γ-position of S unit), but they are also found to acylate to polysaccharides (Petrik et al., 2014). The accumulation of *p*CA indicating lignin deposition level in plants is likely one reason for recalcitrance (Taboada et al., 2010). The *p*CA content of alkaline hydrogen peroxide pretreated grasses, proportional to lignin content, was negatively related with enzymatic digestibility (Li et al., 2012). A study of genetically modified maize lines with similar lignin level (12–15%) demonstrated that the *in vivo* cell wall digestibility is also negatively correlated with the esterified *p*CA and lignin content (Zhang et al., 2011). Due to the acylation with hemicellulose (arabinoxylan), removal of *p*CA together with xylan in sugar cane bagasse increased cellulose accessibility thereof saccharification efficiency (Martínez et al., 2016).

On the other hand, hydroxycinnamates bearing readily cleavable ester linkages was incorporated into lignification to reduce biomass recalcitrance (Ralph, 2010). Incorporation of hydroxycinnamates conjugate—rosmarinic acid, with monolignols into maize cell walls via artificial lignification had remarkably enhanced alkali extractability and digestibility of cell walls (Tobimatsu et al., 2012). More recently, by introducing monolignol ferulate into lignin monomer pool, the engineered poplar demonstrated an improved cell wall digestibility after alkaline pretreatment (Wilkerson et al., 2014).

## Lignin hydroxyl and carboxylic groups

As a major driving force in lignin-enzyme interactions, the hydrophobicity of lignin changes upon the content of hydroxyl and carboxylic groups, which has important influence on enzymatic hydrolysis. Generally, lignin hydrophobicity increases with phenolic hydroxyl (Ar-OH) and decreases with aliphatic OH content. High Ar-OH and low aliphatic OH amounts in lignin were related with higher cellulases adsorption affinity and binding strength (Yu et al., 2014; Huang et al., 2016). Lignin with increased Ar-OH had higher enzyme affinity thereof more inhibition to the hydrolysis of Avicel (Rahikainen et al., 2013; Guo et al., 2014). Yang and Pan recently found that Ar-OH (3.5–4.4 mmol/g lignin) blocked by hydroxypropylation had negligible effect on enzyme adsorption, however, reduced lignin inhibition significantly, which suggested Ar-OH was more related with other interaction for inhibitory effect (Yang and Pan, 2016). Carboxylic groups (COOH) in lignin alter the physicochemical effects of lignin by (i) increasing hydrophilicity; (ii) creating electrostatic charge; and (iii) enhancing hydrogen bond, which could impact lignin- enzyme interactions differently (Nakagame et al., 2011a). Increased COOH in lignin enhanced the hydrolysis efficiency of Avicel as COOH may alleviate the non-productive binding by increasing hydrophilicity and electrostatic repulsion force of lignin to enzymes (Nakagame et al., 2011b; Moxley et al., 2012). Increasing the hydrophilicity of lignin via carboxylation and sulfonation reduced inhibitory effect substantially (Yang and Pan, 2016). However, the correlation of Ar-OH and COOH with enzymatic hydrolysis becomes complicated when different botanical origins and pretreatment methods are used due to the complexity of substrates. For example, high Ar-OH was related with increased sugar yield of Kraft pretreated hardwood and acid pretreated corn (Moxley et al., 2012; Santos et al., 2012), while inconsistent correlations between COOH and enzyme adsorption have been reported on lignins from different botanical origins (Rahikainen et al., 2013; Guo et al., 2014; Yu et al., 2014).

## Other lignin associated factors

**Figure 2:**
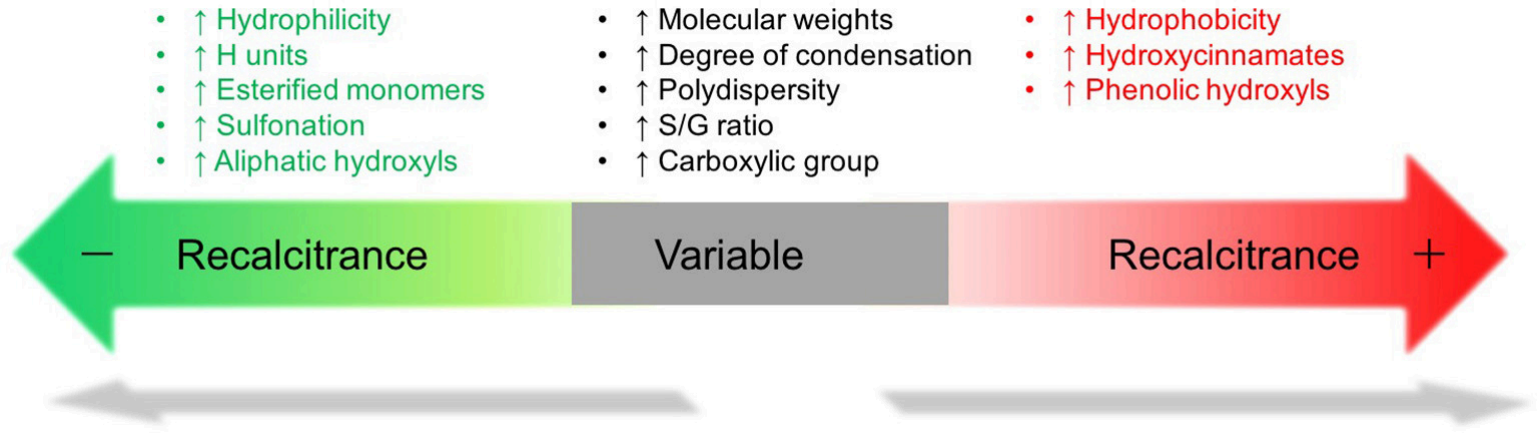
**Schematic relationship of lignin properties to biomass cell wall recalcitrance**.

Other factors of lignin such as molecular weight and polydispersity were found associated with biomass recalcitrance but the correlation is inconclusive (Figure 2). The molecular weight of alkali lignin and lignosulfonate had an opposite effect on enzymatic hydrolysis (Zhou et al., 2013; Li Y. et al., 2016) and no clear impact of the molecular weights on enzyme adsorption was found (Pareek et al., 2013; Guo et al., 2014). Although earlier study indicated that lignin with lower polydispersity thereby higher plasticity, interacts favorably with proteins (Berlin et al., 2006), a few recent reports had difficulty to correlate lignin polydispersity with saccharification/enzyme adsorption (Pareek et al., 2013; Guo et al., 2014; Yang and Pan, 2016).

An alternative approach to enhance saccharification is to manipulate lignin biosynthesis pathway in plant for reduced recalcitrance (Phitsuwan et al., 2013). A few strategies used for lignin biosynthesis perturbation are (i) decreasing lignin content; (ii) relocating lignin deposition; (iii) altering lignin subunits; and (iv) modification of lignin backbone and linkages with carbohydrates (Bonawitz and Chapple, 2010; Simmons et al., 2010; Cesarino et al., 2012). Although the engineered plants' agronomical performances are challenged, enhanced cell wall deconstruction have been found in down-regulation of *CCR* and *CCoAOMT* in maize (Park et al., 2012; Li et al., 2013), *CAD, 4CL* and *COMT* in switchgrass (Fu et al., 2011a,b; Xu et al., 2011), *HCT* in *Arabidopsis* (Gallego-Giraldo et al., 2011), *C3*′*H* and *C4H* in *Eucalyptu*s (Sykes et al., 2015). In addition, inclusion of new lignin precursors has also been used to facilitate lignin depolymerization thereby reduce biomass recalcitrance. With incorporation of several flavonoids and gallate derivatives with easily cleavable ester bonds, the artificially lignified cell wall had enhanced delignification, ruminal and/or enzymatic digestibility and fermentability (Grabber et al., 2010; Elumalai et al., 2012).

## Summary and perspectives

Although the study of lignin's influence on cell wall digestibility has been advanced recently, the correlation of lignin structure and biomass recalcitrance remain complicated due to lignin structural variety and cell wall complexity. According to these studies, the physicochemical properties of lignin are strongly related with recalcitrance (Figure 2). It should be noted that this figure only represents the major findings in certain case and it cannot be solely used as a metric to evaluate biomass recalcitrance as these factors and other unknown factors usually contribute together to cell wall recalcitrance. The impact of lignin on biomass recalcitrance could be very different depending upon plant species and pretreatment techniques. To better understand the cause-effect relation between one property of lignin and biomass recalcitrance, it is desirable to minimize the side effects from other cell wall properties and lignin properties.

## Author contributions

AR conceived the review and reviewed it for technical merit. ML and YP shared in writing the article.

## Disclosure

This manuscript has been authored by UT-Battelle, LLC under contract no. DE-AC05-00OR22725 with the U.S. Department of Energy. The publisher, by accepting the article for publication, acknowledges that the United States Government retains a non-exclusive, paid-up, irrevocable, world-wide license to publish or reproduce the published form of this manuscript, or allow others to do so, for United States Government purposes. The Department of Energy will provide public access to these results of federally sponsored research in Accordance with the DOE Public Access Plan (http://energy.gov/downloads/doe-public-access-plan.

### Conflict of interest statement

The authors declare that the research was conducted in the absence of any commercial or financial relationships that could be construed as a potential conflict of interest.

## Abbreviations

AFEX: ammonia fiber expansion
*4CL*: 4-coumarate:coenzyme A ligase
*C3*′ *H*: *p*-coumaroyl quinate/shikimate 3′-hydroxylase
*C4H*: cinnamate 4-hydroxylase
*CAD*: cinnamyl alcohol dehydrogenase
*CCR*: cinnamoyl-coenzyme A reductase
*CCoAOMT*: caffeoyl-CoA O-methyltransferase
*COMT*: caffeic acid O –methyltransferase
DAP: dilute acid pretreatment
*HCT*: hydroxycinnamoyl CoA: shikimate hydroxycinnamoyl transferase
HWP: hot water pretreatment
SEP: steam explosion pretreatment.
